# Supplementary material for: Navigating Nutrition Beyond Elite Sport: A Qualitative Exploration of Experiences After Retirement
Source: Nutrients. 2025 Dec 15;17(24):3920. doi: 10.3390/nu17243920 (PMC12735450; doi:10.3390/nu17243920)
Supplement: Supplementary file 1 [file nutrients-17-03920-s001.zip › nutrients-4027953-supplementary.pdf]

Supplementary materials

Figure S1. Participant classification framework

	TIER 0: SEDENTARY (NON-ATHLETE)	TIER 1: RECREATIONALLY ACTIVE (NON-ATHLETE)	TIER 2: TRAINED/ DEVELOPMENTAL (ATHLETE)	TIER 3: HIGHLY TRAINED/ NATIONAL LEVEL (ATHLETE)	TIER 4: ELITE/ INTERNATIONAL LEVEL (ATHLETE)	TIER 5: WORLD CLASS (ATHLETE)
PHYSICAL ACTIVITY LEVEL	Do not meet WHO minimum physical activity guidelines*.	Meet WHO minimum physical activity guidelines*.	Regularly training ~3x/week	Completing structured & periodized training. Developing towards (within 20%) of maximal or nearly maximal norms within given sport**.	Maximal, or nearly maximal training, within the given sport norms**, with intention to complete at top-level competition.	Maximal or nearly maximal training, within the given sport norms**
TRAINING	n/a	n/a	Training with purpose to compete.			
SPORT/ ACTIVITY TYPE(S)	e.g. walking, household cleaning	May participate in multiple sports or forms of activity	Identify with a specific sport	Identify with a specific sport	Identify with a specific sport	Identify with a specific sport
CALIBRE	n/a	n/a	Limited skill development	Developing proficiency in sport-specific skills	Highly proficient in sport-specific skills	Exceptional sport-specific skill-level achieved
	Not competing	Not competing	Local-level representation	National-level representation	International-level representation	Olympic & world-medalists
	n/a	n/a	n/a	Achievement within 20% of world-record and/or world-leading performance	Achievement of within 7% of world-record and/or world-leading performance. Top 4–300 in world rankings.	Achievement within 2% of world-record and/or world-leading performance. Top 3–20 in world rankings and/or top 3–10 at an Olympics/World Championships. Top players within top teams [illegible]

Adapted from McKay AKA, Stellingwerff T, Smith ES, et al.; Defining Training and Performance Caliber: A Participant Classification Framework. Int J Sports Physiol Perform 2022; 17(2):317-331.
